# Molecular Mechanisms for Coping with Al Toxicity in Plants

**DOI:** 10.3390/ijms20071551

**Published:** 2019-03-28

**Authors:** Xiang Zhang, Yan Long, Jingjing Huang, Jixing Xia

**Affiliations:** State Key Laboratory for Conservation and Utilization of Subtropical Agro-bioresources, College of Life Science and Technology, Guangxi University, Nanning 530004, China; 1708304025@st.gxu.edu.cn (X.Z.); 1808301038@st.gxu.edu.cn (Y.L.); 1808301015@st.gxu.edu.cn (J.H.)

**Keywords:** aluminum, Al toxicity, Al exclusion, Al tolerance, Al transporter

## Abstract

Aluminum (Al) toxicity is one of the major constraints to agricultural production in acid soils. Molecular mechanisms of coping with Al toxicity have now been investigated in a range of plant species. Two main mechanisms of Al tolerance in plants are Al exclusion from the roots and the ability to tolerate Al in the roots. This review focuses on the recent discovery of novel genes and mechanisms that confer Al tolerance in plants and summarizes our understanding of the physiological, genetic, and molecular basis for plant Al tolerance. We hope this review will provide a theoretical basis for the genetic improvement of Al tolerance in plants.

## 1. Introduction

Aluminum (Al) is the third most abundant element in the earth crust, after Oxygen and Silicon, and the most plentiful metallic element in soil. Al is considered non-toxic to plants when located in near-neutral or alkalescent soil. However, natural processes or human activities can lead to soil acidification (pH < 5.5), the production of Al ions (mainly Al^3+^) from Al oxides, and phytotoxicity. Over 30% of the land and over 40% of the potential arable land are acidic [1], most of which is distributed in the tropics and subtropics, in which important commercial and food crops are planted. Al toxicity limits crop yields by inhibiting root growth and influencing the absorption of water and nutrients [1]. A number of studies have described Al targets in root cells with perturbations to the cell wall, plasma membrane, mitochondria, cytoskeleton, and nucleus [2,3,4,5,6]. Thus, it influences an array of cellular processes including reactive oxygen species (ROS) generation, signal transduction disorder, and lower cell wall extensibility, thereby impairing plant growth [2,3,4,5,6,7,8,9,10,11,12].

**Figure 1:**
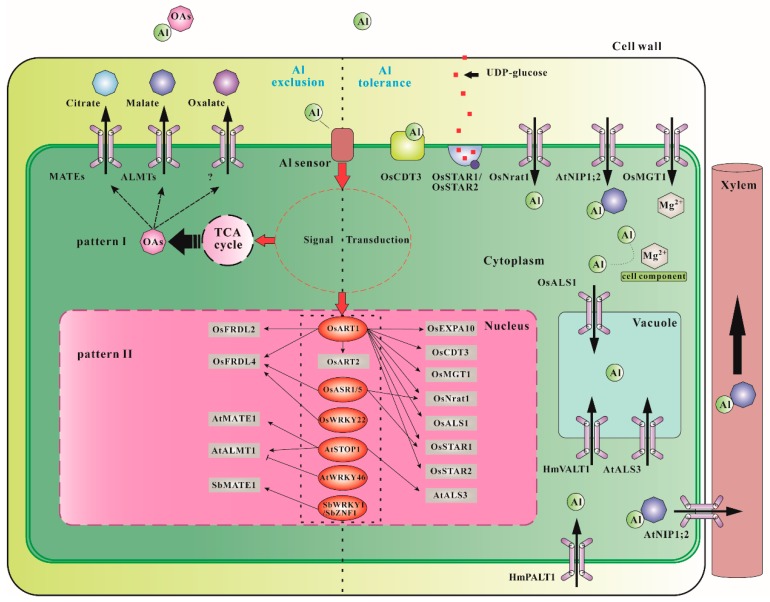
Mechanisms of Al tolerance in plants. MATEs, multidrug and toxic compound extrusion for citrate transport; ALMTs, aluminum-activated malate transporters; OsCDT3, a small peptide with rich cysteine; OsSTAR1/OsSTAR2, UDP-glucose transporter; OsEXPA10, an expansin gene; OsNrat1, plasma membrane-localized Al transporter; AtNIP1;2, plasma membrane-localized Al-malate transporter; AtALS3, plasma membrane-localized Al transporter; HmPALT1, plasma membrane-localized Al transporter; OsMGT1, magnesium transporter; OsALS1, tonoplast-localized Al transporter; HmVALT1, tonoplast-localized Al transporter; OsFRDL2/4, citrate transporter; AtMATE1, citrate transporter; AtALMT1, malate transporter; OsASR1/5, ABA-stress and ripening 1/5; OsART1/2, C_2_H_2_-type zinc finger transcription factor; AtSTOP1, C2H2-type zinc finger transcription factor; OsWRKY22, WRKY transcription factor; AtWRKY46, WRKY transcription factor; SbWRKY1, WRKY transcription factor; SbZNF1, DHHC-type zinc finger transcription factor; SbMATE1, citrate transporter.

To deal with Al toxicity, plants possess several strategies to reduce the noxious consequences of Al toxicity during the evolutionary process. These mechanisms can be divided into (i) Al exclusion mechanisms that prevent Al entering root cells (both apoplastic and symplastic pathway) by secreting organic acid (citrate, malate, and oxalate) to chelate Al cations and (ii) Al tolerance mechanisms in which Al enters root cells and is detoxified and sequestered into vacuoles or other organelles in roots and/or shoots (Figure 1) [5,6,13,14].

Several reviews have described the research progress made on molecular mechanism of Al tolerance in gramineous plants or crops several years ago [12,13,14]. In recent years, a number of new Al-tolerant genes have been identified by using different approaches in higher plants. In the present review, we provide brief summaries on recent discoveries of new genes and molecular mechanisms by which plants cope with Al toxicity. In addition, we provide a perspective on future researches and challenges of the molecular mechanism of Al tolerance and its application for enhancing crop Al tolerance and improving crop yields on acid soils.

## 2. Al Exclusion Mechanisms

It is well-known that root apex is the critical region of Al toxicity. Many plants secrete organic acid (malate, citrate, and oxalate) from the root tip in response to Al stress, which prevents trivalent Al ion from entering root tip cells. Two transporter families that confer Al tolerance through the secretion of organic acids have been identified in plants (Table 1). These include ALMT (aluminum-activated malate transporter) and MATE (Multidrug and toxic compound extrusion). The first identified malate efflux gene was *TaALMT1* from wheat [15]. This protein is a membrane-localized transporter that participates in the efflux of malate from roots in response to Al stress. Homologs of TaALMT1 have been found in other species such as *Arabidopsis*, rape, maize, soybean, rye, *Medicago sativa*, *Holcus lanatus*, and rice [16,17,18,19,20,21,22,23,24,25,26]. Among these homologs, AtALMT1, BnALMT1/2, ScALMT1, GmALMT1, ScALMT1, MsALMT1, and HlALMT1 share similar functions to TaALMT1, which is involved in Al tolerance by mediating the secretion of organic acids. Other identified homologs are found to be unrelated to Al tolerance and involved in other functions. For example, AtALMT4, AtALMT6, AtALMT9, and AtALMT12 in *Arabidopsis* and HvALMT1 in barley are expressed in guard cells and participate in the regulation of the stomatal aperture through anion transport [19,27,28,29,30,31,32]. In maize, ZmALMT1 is localized at the plasma membrane and transports inorganic anions rather than malate, while ZmALMT2 is permeable for malate, citrate, Cl^−^ and NO_3_^−^ [23,24]. Both ZmALMT1 and ZmALMT2 are involved in anion homeostasis. Recently, OsALMT4 is showed to be a malate permeable anions channel, which plays an important role in the growth of rice in low-light environments [33]. More and more studies indicate that ALMTs have multiple functions in plants, including toxic metal resistance, ion homeostasis, mineral nutrition, turgor regulation, pollen tube growth, and guard cell regulation [34]. Another organic acid efflux transporter family MATEs have been identified in barley (HvAACT1), sorghum (SbMATE1), wheat (TaMATE1), *Arabidopsis* (AtMATE1), rye (ScFRDL2), maize (ZmMATE1), and rice (OsFRDL2 and OsFRDL4) [35,36,37,38,39,40,41,42]. They function as a plasma membrane efflux transporter and are responsible for the Al-activated citrate release. In addition to citrate and malate, buckwheat has been shown to secrete oxalate to chelate Al in the rhizosphere, but transporters for oxalate release in respond to Al have not been identified [43,44,45].

In response to Al stress, organic acid is secreted from the root cells to chelate Al in two distinct patterns [74,75]. In the first pattern, no discernible delay can be observed between the initiation of organic acid secretion and Al addition in barley and wheat [76,77]. In the second pattern, an obvious delay (several hours to days) between Al exposure and the secretion of organic acid is observed in sorghum, rye, maize, *Arabidopsis*, and rice [17,35,37,40,42]. The rapid secretion of organic acid following exposure to Al in the first pattern suggests that organic acid secretion does not require the induction of genes encoding organic acid transporters such as TaALMT1 and HvAACT1. The presence of external Al enables and/or enhances organic acid release from the root cells. However, in the second pattern, a distinct lag between Al exposure and the secretion of organic acids, and/or the expression of organic acid transporters are observed. Three possibilities have been proposed to explain the variable patterns [78]: (i) External Al directly interacts with the transporter, leading to its conformational change and the increase of its mean conductance or open time. This is analogous to ligand-gated channels. (ii) Al binds to specific membrane receptors through the signal transduction pathways in the cytoplasm to regulate the channel activity. (iii) Al enters the cytoplasm and directly or indirectly activates or enhances the activity of organic acid transporters. It has recently been shown that Al enhances the activity of organic acid transporters. The transport activity of TaALMT1, AtAMLT1, OsFRDL4, and HvAACT1 is enhanced by exogenous Al in Xenopus oocytes [17,38,40,79]. To date, researchers have mainly focused on the mechanism underlying Al activation of ALMTs activity. ALMT1 protein is composed of an N-terminus region containing six transmembrane domains and a variable long hydrophilic C terminus [30]. Three main residues (Glu274, Asp275, and Glu284) in C terminus of TaALMT1 are shown to be required for Al-dependent transport enhancement [80]. However, Ligaba et al. [81] reported that both N- and C-terminus regions are involved in Al enhancement of transport activity. Recently, ALMT activity has also been found to be negatively regulated by c-aminobutyric acid (GABA) [82]. Sequence analysis suggested that ALMTs contain a GABA-binding motif with homology to the one presented in the GABA_A_ receptor, which plays a critical role in the relationship between malate efflux and endogenous GABA concentrations, as well as in GABA transport by ALMTs [82,83]. However, the regulatory mechanism of ALMT1 transport activity by Al and GABA is still poorly understood. The crystal structural studies of ALMT1 will be required to understand the conformational changes of the ALMTs upon interaction with Al or GABA and the underlying mechanism.

Not only organic acids chelate Al. Other organic compounds are also reported to have the capacity to chelate trivalent Al ions, such as phenolics and benzoxazinoids. Kidd et al. [84] found that maize secretes the phenolic compounds catechol, catechin, and quercetin to chelate Al. Hydroxide radicals in these phenolic compounds provide potential targets for Al ion chelation, although phenolic compounds are less effective chelators compared to organic acids [4]. Recently, Al- and salicylic-acid-activated root efflux of benzoxazinoids (HA) has been demonstrated to chelate Al and enhance Al tolerance in maize [85]. However, genes involved in Al-induced secretion of phenolics and benzoxazinoids have not been identified in any plant species yet.

The increase in rhizosphere pH is another Al exclusion mechanism in plants. Elevation in the rhizosphere pH can lower the solubility, activity, and potential toxicity of Al, thus achieving higher tolerance to Al for plants [86,87,88]. For instance, an Al-tolerant mutant *alr-104* exhibits an Al-induced increase in rhizosphere pH through the increased H^+^ influx in *Arabidopsis* [87]. In other plant species (wheat, buckwheat), the Al-tolerant lines can maintain a relatively higher pH surrounding the root apex than Al-sensitive lines [44,86]. PM H^+^-ATPase has been shown to be involved in regulating rhizosphere pH [86,87,88]. Additionally, the secretion of organic acids from the root apex to the rhizosphere can also modify rhizosphere pH in the presence of Al [88].

## 3. Al Tolerance Mechanisms

Trivalent Al ions display severe phytotoxicity to many cereal crops (rice, wheat, maize, buckwheat, and rye), and micromolar levels of Al rapidly inhibit root elongation. Once Al enters the plant, highly Al-tolerant cereal species employ multiple genes and mechanisms to be involved in Al tolerance at different levels in the roots. To date, Al tolerance mechanisms mainly include cell wall modification, the uptake and subsequent sequestration of Al, and root-to-shoot translocation of Al [14].

Considering that the cell wall contains most Al amongst the whole roots [7,89,90], it is reasonable that plants cope with Al stress through cell wall modifications. The primary root cell wall is mainly composed of cellulose, pectins, and hemicelluloses [91]. Cellulose is made up of unbranched 1,4 β–D glucan chains and is considered not to bind Al, while both pectins with the negatively charged carboxyl groups and hemicelluloses with highly branched structures can interact with Al^3+^ ions [91,92]. The negatively charged carboxyl groups of pectins can crosslink with Ca^2+^ ions. However, trivalent Al^3+^ ions have stronger binding to pectins than Ca^2+^. Under Al stress, replacement of pectin-bound Ca^2+^ ions by Al makes the cell wall thicker and more rigid, thus inhibiting cell extension and division [93]. The highly methylated pectins by pectin methylesterases (PMEs) are converted to negatively charged demethylation form, leading to more Al binding to pectins. Al-tolerant cultivars exhibiting a higher methylated pectin proportion and lower PME activity are found in rice, maize, and buckwheat [89,92,94]. Furthermore, *OsPME14*-overexpressed lines showed more Al accumulation in root tip cell wall and increased sensitivity to Al [95]. On the other hand, hemicelluloses were also shown to be a major site for Al accumulation, significantly contributing to Al adsorption and root growth in *Arabidopsis* [92]. The hemicellulose (xyloglucan)–cellulose network is catalyzed by xyloglucan endohydrolase (XEH) and xyloglucan endotransglucosylase (XET) encoded by xyloglucan endotransglucosylase-hydrolase (XTH) genes, which are involved in cell expansion by cutting and rejoining of xyloglucan chains. XTH gene expression and enzyme activity are influenced by hormonal and environmental stimuli [91]. Plant hormones such as auxins and gibberellins can increase the expression and activity of XTH while Al represses it [50,96,97]. Zhu et al. [50] reported that Al inhibited the expression and activity of XTH31 in *Arabidopsis* roots and mutation of *XTH31* enhanced Al tolerance by reducing cell wall xyloglucan content and lowering its Al binding capacity. In rice, *OsSTAR1* (for sensitive to Al rhizotoxicity 1) encodes a nucleotide binding domain, while *OsSTAR2* encodes a transmembrane domain. OsSTAR1 and OsSTAR2 together form a complex that functions as a vesicle membrane-localized ABC transporter in roots and transports UDP-glucose into the cell wall, which may modify the cell wall and mask the sites through which Al can bind, reducing Al accumulation and damage in the cell wall [67].

In plants, several types of transporters have been shown to be involved in the uptake and subsequent sequestration and root-to-shoot translocation of Al in plants (Table 1). In rice, *OsNrat1* (Nramp aluminum transporter 1) encodes a plasma membrane-localized transporter belonging to the Nramp (natural resistance-associated macrophage protein) family, showing low similarity to other Nramp members. OsNrat1 does not transport divalent metals (Mn^2+^, Fe^2+^, Cd^2+^) as other Nramp members, but specifically transports trivalent Al ions and plays an important role in rice Al tolerance [66]. Bioinformatic and functional analysis identify Ala-Ile-Ile-Thr motifs as a key determinant of Al selectivity for Nrat1 [98]. On the other hand, OsALS1 is a tonoplast-localized ATP-binding cassette (ABC) transporter responsible for sequestrating Al into the vacuoles in the roots [57]. Knockout of *OsALS1* in rice led to significant hypersensitivity to Al, suggesting that OsALS1 is required for rice internal Al detoxification. Homologs of OsALS1 (FeALS1.1 and FeALS1.2) in buckwheat have also been identified, which are implicated in vacuolar sequestration of Al [52]. Since OsNrat1 and OsALS1 share similar expression and localization patterns, these two proteins function cooperatively; i.e., Nrat1 mediates cell entry of Al, and Al is sequestered into vacuoles by OsALS1, thus mediating Al detoxification in rice roots [57,66]. In *Arabidopsis*, AtALS3 (Aluminum sensitive 3) is an ABC-transporter primarily expressed in the phloem of roots, leaves, stems, and flowers [46]. AtALS3 is suggested to function as a Al transporter and redistribute accumulated Al away from sensitive tissues to protect the sensitive root tip from Al toxicity. Recently, two members of the aquaporin (AQP) family in *Hydrangea macrophylla*, *HmPALT1*, and *HmVALT1* have been reported to encode plasma membrane and vacuolar Al transporters, respectively [55]. Although the Al form transported by these two transporters remains unclear, they facilitate to transport Al into the cytosol and vacuoles in *Hydrangea*. More recently, NIP1;2, the closest homolog of HmPALT1, was identified as a bidirectional Al-malate transporter functioning in Al removal from root cell walls and root-to-shoot Al translocation in *Arabidopsis* [47]. Furthermore, Al-malate transport of NIP1;2 is dependent on Al-activated malate release mediated by AtALMT1 in roots. Hence, a coordinated operation between external and internal Al detoxification mechanisms was linked by NIP1;2 and AtALMT1.

In comparison to most plant species such as rice and *Arabidopsis*, a small fraction of plant species, including buckwheat, *hydrangea*, tea, and *melastoma malabatbricum*, are able to translocate and accumulate Al at a high level in aerial parts without showing obvious toxicity symptoms [45,99,100,101,102]. Some studies have revealed that small organic compounds play key roles in the uptake, translocation, accumulation, and internal detoxification of Al in these Al-accumulating plants. For example, in buckwheat, Al is taken up in the ionic form and chelated with internal oxalate in root cells, forming a nontoxic Al-oxalate complex at a 1:3 ratio. During the root-to-shoot Al translocation, Al-oxalate (1:3) is converted to Al-citrate (1:1) in the xylem. When Al-citrate is transported into the vacuoles of the leaf cells, Al-citrate is changed back to Al-oxalate [45,99]. In hydrangea, Al is found to be complexed with delphinidin 3-glucoside and 3-caffeoylquinic in the sepals and with citrate at a 1:1 ratio in the leaves [100]. Al is also demonstrated to be chelated with catechin in tea leaves [103]. These studies indicate that internal detoxification of Al in the Al-accumulating plants is achieved by formation of nonphytotoxic Al complexes with small organic compounds and by sequestrating Al into the vacuoles of the leaf cells.

Direct Al transport is not the only way to mediate Al detoxification. It has been shown that the transport of Mg could also relieve Al phytotoxicity. OsMGT1 (Magnesium transporter 1) is a plasma membrane-localized transporter for Mg in rice. Chen et al. [65] found that knockout of this gene significantly decreased Mg uptake and increased the sensitivity to Al in rice, and an Al-sensitive phenotype in knockout lines under Al treatment could be recovered by exogenous micromolar Mg supply. One hypothesis is that the increased Mg concentration in the cytosol contributes to the competitive inhibition of potential targets of Al such as DNA, RNA, ATP, inorganic phosphate, proteins, and other cellular components [65]. This finding proposed a possible mechanism for plants to detoxify internal Al by means of competitive inhibition with increasing Mg uptake into the cells.

## 4. Transcriptional Regulation of Al Tolerance Genes

Transcription factors like zinc finger protein, MYB, WRKY, NAC, and bZIP families act as the early responders to environmental signals and modulate the expression of downstream genes that are required for plants to adapt to abiotic stresses [104]. Two kinds of transcription factors, C_2_H_2_-type zinc finger protein and WRKY, were reported to play important roles in regulating Al tolerance gene expression (Table 1). In *Arabidopsis*, AtSTOP1 belongs to the C_2_H_2_-type zinc finger transcription factor family functioning in both proton and Al tolerance [48]. The expression of *AtSTOP1* was unaffected by exogenous Al, but the expression of several Al-tolerance genes regulated by AtSTOP1, including *AtALS3*, *AtALMT1*, and *AtMATE1*, was induced by Al [46,105,106]. Therefore, the involvement of AtSTOP1 in Al induction of gene expression might be regulated by Al at posttranscriptional or posttranslational levels. Recently, an F-box protein, RAE1, was found to regulate AtSTOP1 stability, which can interact with and regulate AtSTOP1 degradation via the ubiquitin–26S proteasome pathway [107].

OsART1, a homolog of AtSTOP1, plays a similar function in rice [58]. It is localized to the nucleus and is constitutively expressed in all root cells with or without Al treatment. But unlike AtSTOP1, it specifically functions in Al tolerance. Microarray analysis suggested that at least 31 Al-responsive genes are regulated by OsART1, which are involved in both internal and external detoxification of Al in rice. Among them, ten genes have been functionally characterized. These included *OsSTAR1*, *OsSTAR2*, *OsNrat1*, *OsALS1*, *OsMGT1*, *OsFRDL4*, and *OsFRDL2*, which are discussed above as rice Al tolerance genes [39,40,65,66,67,98]. The other three genes are *OsCDT3*, *OsEXPA10,* and *OsART2* [59,62,63]. OsCDT3 is a small cysteine-rich peptide localized on the plasma membrane and may prevent Al entry into root cells through direct Al binding, therefore, contributing to rice Al tolerance [62]. OsEXPA10 is one of the expansin genes up-regulated by Al and is involved in normal root cell elongation, but its contribution to Al tolerance is small [63]. OsART2, a homolog of OsART1, is highly induced by Al. The knockout of *OsART2* resulted in increased sensitivity to Al in rice [59]. However, OsART2 contributes more modestly to Al tolerance than OsART1. RNA-sequencing analysis showed that four genes implicated in Al tolerance are regulated by OsART2 and do not overlap with the genes regulated by OsART1, indicating that OsART1 and OsART2 regulate different pathways involved in rice Al tolerance.

Recently, homologs of OsART1/AtSTOP1 have also been identified in other plant species, such as in Yorkshire fog (HlART1), wheat (TaSTOP1), tobacco (NtSTOP1), rice bean (VuSTOP1), pigeon pea (CcSTOP1), soybean (GmSTOP1), and sorghum (SbSTOP1) [25,51,53,54,69,72,73]. These OsART1/AtSTOP1-like proteins from different species or even from one species play different roles in Al and H^+^ tolerance, although these proteins possessed a highly conserved Cys_2_His_2_ zinc finger domain. For example, NtSTOP1 is involved in both Al tolerance and proton tolerance in tobacco while VnSTOP1 only functions in proton tolerance in rice bean. In soybean, all three GmSTOP1s function in proton tolerance, but only GmSTOP1-1 and GmSTOP1-3 regulate Al tolerance. The exact mechanism for such different roles requires further investigations.

In higher plants, the WRKY transcription factor is characterized by the presence of specific WRKY domains that bind to W-box sequences ((T/C) TGAC (T/C)) in the promoter region of the candidate regulated genes. In *Arabidopsis*, AtWRKY46, a member of the WRKY transcription factor family, was reported to function as a transcriptional repressor of AtALMT1 [49]. The expression of AtWRKY46 colocalizes with that of AtALMT1 in roots. Under Al stress, *AtWRKY46* expression is decreased while *AtALMT1* expression is increased. Mutation of *AtWRKY46* promotes the expression of AtALMT1, root malate secretion, and Al tolerance. Furthermore, AtWRKY46 binds to W-box sequences in the *AtWRKY46* promoter. These findings indicate that AtWRKY46 is a negative regulator of *AtALMT1* expression. Recently, OsWRKY22, another member of the WRKY transcription factor family, has been identified to be involved in Al tolerance in rice [68]. In contrast to AtWRKY46, OsWRKY22 is a positive regulator of rice Al tolerance. In response to Al, OsWRKY22 promotes Al-induced *OsFRDL4* expression by binding to W-box *cis* elements within the promoter of *OsFRDL4*, resulting in enhancing Al-induced citrate secretion and Al tolerance in rice. Furthermore, OsWRKY22 cooperates with ART1 to regulate Al-induced expression of OsFRDL4 and citrate secretion. More recently, both SbWRKY1 and a DHHC-type zinc finger transcription SbZNF1 are reported to *trans*-activate *SbMATE* expression through binding to MITE repeat *cis* elements within *SbMATE* promoter in sorghum [70]. Interestingly, *SbMATE* expression is highly correlated with sorghum Al tolerance, which is found to be regulated by both the number of MITE repeats and the expression level of *SbWRKY1* and *SbZNF1*. These findings provide a potential strategy for improving plant Al tolerance in acidic soils by means of finetuning the concerted *cis-trans* interactions.

In addition to OsART1/AtSTOP1 and WRKY transcription factor, two ASR genes (ABA-stress and ripening), *OsASR1* and *OsASR5*, have also been proposed to be involved in Al tolerance in rice [60,61,108,109]. Both OsASR1 and OsASR5 are localized in the cytoplasm and nucleus and function as transcription factors to regulate *OsSTAR1* expression through binding to its promoter region. They have compensatory and complementary functions in Al tolerance. However, down-regulation of *OsASR5* by RNAi also decreased tolerance to drought stress and displayed some morphologic changes, indicating that OsASR5 has other potential roles in environmental stresses and plant development.

Other studies regarding the transcriptional regulation of Al tolerance genes focused on alterations in the promoter region and copy number of genes. In wheat, the constitutively high expression of *TaALMT1* in Al-tolerant lines is associated with duplicated and triplicated tandem repeats in the promoter region [110]. In sorghum, the higher *SbMATE1* expression is associated with more repeat number of MITEs (tourist-like miniature inverted repeat transposable elements) in the *SbMATE1* promoter [37]. A transposable element insertion in the promoter of MATE/AACT genes is also found to enhance their expression in barley (*HvAACT1*), wheat (*TaMATE1B*), and rice (*OsFRDL4*) [64,71,111]. In Yorkshire fog *(Holcus lanatus*), higher *HlALMT1* expression in an Al-tolerant accession is attributed to an increased number of HlART1 cis-acting elements in its promoter [25]. Additionally, the elevated expression for *ZmALMT1* in maize and *ScALMT1* in rye is caused by their increased copy number in the genome [18,112]. These findings revealed that different mechanisms are involved in regulating the expression of *ALMT* and *MATE*, including increased tandem repeated elements, transposable element insertion, increased copy number, and increased number of cis-acting elements.

## 5. MicroRNA Mediates Al Tolerance

MicroRNAs (miRNA) are small non-coding RNAs with an important role in regulating gene expression by silencing their complementary mRNA targets and translational repression, which participate in numerous biological processes including development, metabolism, and biotic and abiotic stress responses in higher plants. Several studies have shown that miRNAs are involved in the plant response to Al stress. Lima et al. [113] reported that miRNA168, miRNA528, and miRNA399 are up-regulated in rice roots of both *japonica* and *indica* varieties under Al stress while only miRNA395 is down-regulated. In wild soybean, 30 miRNAs were identified to be responsive to Al stress [114]. Recently, comparative miRNA expression analysis between Al-tolerant Tibetan wild barley and Al-sensitive cultivated barley identified 50 Al-responsive miRNAs [115]. Among them, some miRNAs, such as miR160, MiR393, and PC-miR1, were found to be exclusively expressed in Al-tolerant Tibetan wild barley and related to Al tolerance. Based on degradome identification and bioinformatics analysis, target genes of the above-mentioned miRNAs in different plant species participated in an array of cellular processes including auxin-response pathways, ROS detoxification, cell wall modification, and nutrient and carbon metabolism. Details on how the miRNAs regulate downstream gene expression involved in Al tolerance requires further investigation in future studies.

## 6. Arbuscular Mycorrhizas Mediates Al Tolerance

The roots of most higher plants are associated with soil microorganisms that directly or indirectly affect plant growth and development. Among these microflora, arbuscular mycorrhizal (AM) fungi are reported to play a key role in enhancing plant tolerance to diverse abiotic stresses in soil, including Al toxicity [116]. For example, Rufyikiri et al. [117] reported that AM fungi could be effective in alleviating Al toxicity to banana plants. Inoculation with AM fungi and saprobe fungi combinations can improve Al tolerance in *Eucalyptus globulus* [118]. Recently, an early colonization with AM fungi is found to be an important factor in protecting wheat from Al toxicity [119]. Previous studies have revealed several possible mechanisms of Al tolerance induced by AM fungi in higher plants, including that (a) AM fungi improve the uptake of phosphorus (P) and other nutrients in their host plants through the interaction Al-P in colonized roots, which is critical in maintaining plant growth under Al stress [116,120,121]; (b) AM fungi stimulate the processing of carbon in roots through the citric acid cycle to enhance the exudation of organic acids, which chelates Al^3+^ in the rhizosphere [116]; (c) glomalin-related soil protein (GRSP) produced by AM fungi has the capacity to sequester Al^3+^ in the rhizosphere [116,122]; (d) AM fungal structures such as spores and hyphae have the capacity to bind Al directly or build an enlarged mycorrhizosphere in which Al is detoxified [123,124,125,126]. Further details on the role of AM fungi in improving Al tolerance in higher plants require investigation in future studies.

## 7. Conclusions and Remarks

Over recent decades, substantial progress has been made in the understanding of physiological and molecular mechanisms of Al tolerance in higher plants. To date, the vast array of genes involved in Al tolerance such as MATE, ALMT, and ABC transporters have been identified in different species (Table 1). Transcription factors that regulate these genes, including ART1/STOPs and WRKYs, have also been identified in different species (Table 1). The mechanisms by which Al regulates these transcription factors are still however unknown. For example, expression of *OsART1* and *AtSTOP1* is not affected by Al but their downstream genes are induced by Al treatment. It will be interesting to investigate how OsART1 and AtSTOP1 are activated to regulate the transcription of Al tolerance genes by Al. Furthermore, how plants cells sense and signal Al from the rhizosphere is still unclear, and the identification of plasma membrane-localized sensor specific responses to Al is required to illuminate the signal transduction pathways intracellularly induced in response to Al toxicity. MicroRNA is a relatively new research area and studies in this area require more focuses to identify the role of miRNAs in gene regulation under Al stress. The rapid development of whole-genome sequencing and the genome-editing technology provides new opportunities for unraveling these mechanisms and identifying novel ones.

Identification of Al tolerance genes and mechanisms makes it possible to breed Al-tolerant crop species and cultivars via molecular breeding and transgenic approaches. To date, *ALMT* and *MATE* genes have been used to improve the Al tolerance in sorghum, barley, and wheat [37,127,128]. However, there are some limitations of the present methods to introduce single genes into Al-sensitive species and cultivars. For instance, barley transformed with *HvAACT1* displayed lower tolerance to Al than Al-tolerant barley cultivar, Dayton [56]. Furthermore, Al tolerance in some crop species is controlled by multiple genes. Therefore, introduction of multiple Al tolerance genes should be developed for enhancing Al tolerance and improving crop yields on acid soils through conventional breeding and modern biotechnology. On the other hand, the use of diverse AM fungal species adapted to a high Al presence should be considered as an important way to alleviate the Al toxicity and increase crop yields in acidic soils.

## Figures and Tables

**Table 1:** The list of Al tolerance genes identified in plants.

Genes	Plant Species	Functions	References
AtALMT1	*Arabidopsis thaliana*	Transport malate	[17]
AtALS3	*Arabidopsis thaliana*	Transport Al away from sensitive tissue	[46]
AtMATE	*Arabidopsis thaliana*	Transport citrate	[36]
AtNIP1;2	*Arabidopsis thaliana*	Transported Al-malate into cytoplasm	[47]
AtSTOP1	*Arabidopsis thaliana*	Regulate Al tolerance genes	[48]
AtWRKY46	*Arabidopsis thaliana*	Regulate Al tolerance genes	[49]
AtXET31	*Arabidopsis thaliana*	Function in cell wall extension	[50]
BnALMT1/2	*Brassica napus*	Transport malate	[16]
CcSTOP1	*Cajanus cajan*	Homolog of AtSTOP1	[51]
FeALS1.1	*Fagopyrum esculentumMoench.*	Homolog of OsALS1	[52]
FeALS1.2	*Fagopyrum esculentumMoench.*	Homolog of OsALS1	[52]
GmALMT1	*Glycine max*	Transport malate	[21]
GmSTOP1-1	*Glycine max*	Homolog of AtSTOP1	[53]
GmSTOP1-1	*Glycine max*	Homolog of AtSTOP1	[53]
GmSTOP1-3	*Glycine max*	Homolog of AtSTOP1	[53]
HlALMT1	*Holcus lanatus*	Transport malate	[54]
HlART1	*Holcus lanatus*	Homolog of OsART1	[25]
HmPALT1	*Hydrangea macrophylla*	Transport Al into the cytoplasm	[55]
HmVALT1	*Hydrangea macrophylla*	Sequester Al into the vacuoles	[55]
HvAACT1	*Hordeum vulgare L.*	Transport citrate	[56]
MsALMT1	*Medicago sativa*	Transport malate	[26]
NtSTOP1	*Nicotiana tabacum*	Homolog of AtSTOP1	[54]
OsALS1	*Oryza sativa*	Sequester Al into the vacuoles	[57]
OsART1	*Oryza sativa*	Regulate Al tolerance genes	[58]
OsART2	*Oryza sativa*	Regulate Al tolerance genes	[59]
OsASR1	*Oryza sativa*	Regulate Al tolerance genes	[60]
OsASR5	*Oryza sativa*	Regulate Al tolerance genes	[61]
OsCDT3	*Oryza sativa*	Bind Al	[62]
OsEXPA10	*Oryza sativa*	Mediate cell wall loosening	[63]
OsFRDL2	*Oryza sativa*	Transport citrate	[39]
OsFRDL4	*Oryza sativa*	Transport citrate	[64]
OsMGT1	*Oryza sativa*	Transport Mg into the cytoplasm	[65]
OsNrat1	*Oryza sativa*	Transport Al into the cytoplasm	[66]
OsSTAR1	*Oryza sativa*	Transport UDP-glucose to cell wall	[67]
OsSTAR2	*Oryza sativa*	Transport UDP-glucose to cell wall	[67]
OsWRKY22	*Oryza sativa*	Regulate Al tolerance genes	[68]
SbMATE1	*Sorghum bicolor L.*	Transport citrate	[37]
SbSTOP1	*Sorghum bicolor L.*	Homolog of AtSTOP1	[69]
SbWRKY1	*Sorghum bicolor L.*	Regulate Al tolerance genes	[70]
SbZNF1	*Sorghum bicolor L.*	Regulate Al tolerance genes	[70]
ScALMT1	*Secale cereale L.*	Transport malate	[18]
ScFRDL2	*Secale cereale L.*	Transport citrate	[35]
TaALMT1	*Triticum aestivum L.*	Transport malate	[15]
TaMATE1	*Triticum aestivum L.*	Transport citrate	[41]
TaMATE1B	*Triticum aestivum L.*	Transport citrate	[71]
TaSTOP1	*Triticum aestivum L.*	Homolog of AtSTOP1	[72]
VuSTOP1	*Vigna umbellata*	Homolog of AtSTOP1	[73]
ZmMATE1	*Zea mays*	Transport citrate	[42]
